# Maternal readiness for weaning: Influencing factors and determinants

**DOI:** 10.1590/1980-220X-REEUSP-2025-0163en

**Published:** 2025-09-26

**Authors:** Kamile Akça, Songül Kekil, Meltem Özcan

**Affiliations:** 1 Gaziantep Islam Science and Technology University, Faculty of Health Sciences, Gaziantep, Türkiye.; 2 Erzincan Binali Yıldırım University, Health Services Vocational School, Erzincan, Türkiye.

**Keywords:** Breastfeeding, Weaning, Maternal Behavior, Infant, Breastfeeding and Complementary Feeding, Aleitamento Materno, Desmame, Comportamento Materno, Lactente, Aleitamento Materno Complementado

## Abstract

**Objective:**

To assess maternal readiness for weaning and examine the socio-demographic and behavioral factors influencing weaning decisions.

**Method:**

A cross-sectional study was conducted at three Family Health Centers located in a provincial center between October 2024 and January 2025. The study included 202 breastfeeding mothers. Data were collected through face-to-face interviews using a sociodemographic questionnaire and the validated Readiness for Weaning Scale. Statistical analyses included t-tests, ANOVA, chi-square tests, and logistic regression.

**Results:**

Mothers with higher education had significantly higher scale scores than those with lower education. Breastfeeding education was associated with increased weaning readiness. Planned pregnancy and maternal perception of milk sufficiency were positively correlated with higher scale scores. Smoking was associated with lower readiness. Logistic regression indicated that maternal perception of milk sufficiency significantly predicted weaning consideration, while lower education levels were associated with reliance on traditional weaning methods. Traditional weaning methods referenced in this study include culturally ingrained practices such as abrupt cessation, use of herbal substances, or application of unpleasant tastes to the breast.

**Conclusion:**

Maternal education, psychological preparedness, and professional support play key roles in structured weaning. Targeted interventions are needed to enhance maternal confidence and informed decision-making in infant feeding transitions. Limitations of the study include its cross-sectional design, which prevents causal inference, and the reliance on self-reported data, which may be subject to recall or social desirability bias.

## RESUMO

**Objetivo:**

Avaliar a prontidão materna para o desmame e examinar fatores sociodemográficos e comportamentais que influenciam essa decisão.

**Métodos:**

Estudo transversal realizado em três Unidades de Saúde da Família de um centro provincial entre outubro de 2024 e janeiro de 2025, com 202 mães lactantes. Os dados foram coletados por entrevistas presenciais utilizando questionário sociodemográfico e a Escala de Prontidão para o Desmame validada. As análises incluíram testes *t*, ANOVA, qui-quadrado e regressão logística.

**Resultados:**

Mães com maior escolaridade apresentaram escores significativamente mais altos. Educação em aleitamento, gravidez planejada e percepção de suficiência de leite correlacionaram-se positivamente com prontidão, enquanto tabagismo mostrou associação negativa. A percepção de suficiência de leite previu a consideração do desmame; menor escolaridade associou-se ao uso de métodos tradicionais.

**Conclusões:**

Escolaridade, preparo psicológico e apoio profissional são essenciais para um desmame estruturado. Intervenções específicas devem fortalecer a confiança materna e a tomada de decisão informada. Limitações desenho transversal e dados autorreferidos podem limitar inferências causais e introduzir viés de recordação ou desejabilidade social.

## INTRODUCTION

Breastfeeding is widely recognized as the optimal method of infant nutrition, offering numerous health benefits for both the mother and the child. The World Health Organization (WHO) recommends exclusive breastfeeding for the first six months of life, followed by continued breastfeeding with complementary foods up to two years or beyond^(1)^. Despite these recommendations, global adherence rates remain suboptimal, with many mothers discontinuing breastfeeding earlier than recommended due to various socio-demographic, psychological, and cultural factors^(2)^. Early weaning can be influenced by multiple factors, including maternal employment, educational attainment, family support, and healthcare guidance^(3)^.

Maternal decision-making regarding weaning is complex and shaped by both individual and environmental factors. Research suggests that higher maternal education is associated with increased breastfeeding duration and informed decision-making about weaning^(4)^. Additionally, prior breastfeeding experience, prenatal education, and social support play significant roles in shaping maternal readiness for weaning^(5,6)^. While some studies highlight the positive influence of professional lactation support and structured breastfeeding education^(7)^, others point to cultural expectations and traditional beliefs as barriers to timely and structured weaning^(8)^. For example, in parts of rural Uganda and Northern Cyprus, mothers frequently use herbal substances, apply bitter-tasting plant extracts to the breast, or rely on sudden cessation to encourage weaning, practices that are culturally accepted but may conflict with evidence-based guidelines^(8,9)^. Such traditional methods are often passed down intergenerationally and reflect deeply held cultural norms, particularly among populations with lower formal education levels.

Despite extensive research on breastfeeding initiation and duration, there is a notable gap in understanding maternal readiness for weaning and the factors influencing this transition. Addressing this gap is crucial for developing targeted interventions that support mothers in making informed and confident weaning choices. Therefore, the objective of this study is to assess maternal readiness for weaning and examine the socio-demographic and behavioral factors influencing weaning decisions.

## METHOD

### Study Design and Setting

This study employed a cross-sectional design and utilized structured questionnaires to assess maternal readiness for weaning. The study was conducted at three Family Health Centers (FHCs) located in a provincial center in eastern Türkiye.

### Population and Sample

The study population consisted of mothers who visited the Family Health Centers (FHCs) for any reason between October 2024 and January 2025. The study sample included mothers who had infants aged 18 months or older and were still breastfeeding at the time of data collection. Additional inclusion criteria were: residence in the province for at least six months, ability to communicate in Turkish, and adequate literacy to comprehend and respond to the study instruments. Exclusion criteria included mothers with visual, hearing, speech, or physical disabilities, psychiatric disorders, multiple births (twins or triplets), or infants with chronic diseases or significant health conditions. The sample size for this study was determined through an a priori power analysis using G*Power 3.1.9.7, considering the primary statistical approach of logistic regression analysis. Since no prior studies have used the Readiness for Weaning Scale (RWS), there were no effect size estimates available. Therefore, we based our calculation on a significance level of *α* = 0.05, power of 0.80, and an estimated medium effect size (Cohen’s f^2^ = 0.15), following standard recommendations in behavioral and health sciences research^(10)^. The power analysis was conducted to detect a significant relationship between socio-demographic factors and maternal readiness for weaning. The analysis indicated that a minimum of 178 participants would be required to achieve sufficient statistical power. Given this requirement, a total of 202 mothers were recruited to ensure a sample size that met the power threshold while allowing for potential data attrition. Furthermore, the sample size met the criterion of at least 10–20 events per predictor variable (EPV) required for stable logistic regression analysis.

### Measures

#### Socio-Demographic Characteristics

The socio-demographic characteristics questionnaire was used to collect detailed information on maternal and infant characteristics relevant to breastfeeding and weaning practices. For the purposes of this study, traditional weaning methods were also assessed. Traditional weaning methods are defined as culturally ingrained practices commonly used in the local context. These include abrupt cessation of breastfeeding, application of herbal substances or bitter-tasting foods to the breast to discourage feeding, and reliance on family traditions or advice from elder relatives (such as grandmothers or mothers-in-law) rather than professional recommendations. These methods are distinct from gradual, structured, or professionally guided weaning approaches.

#### The Readiness for Weaning Scale (RWS)

The Readiness for Weaning Scale (RWS) is a self-report instrument developed to assess maternal readiness for transitioning from breastfeeding^(11)^. The RWS comprises 19 items distributed across four dimensions: weaning intention, infant readiness, weaning technique, and maternal readiness. Each item is rated on a 5-point Likert scale, ranging from 1 (Strongly Disagree) to 5 (Strongly Agree), with higher scores indicating greater maternal readiness for weaning. The total RWS score is obtained by summing all item responses, yielding a range from 19 to 95. Because no universal cut-offs exist, we interpreted scores that were at least one standard deviation above the sample mean as “higher readiness” and those at least one standard deviation below the mean as “lower readiness,” following current psychometric practice. In this study, the scale was used in its original validated Turkish form, developed and psychometrically validated in a Turkish population^(11)^. The validation process included assessment of construct validity and internal consistency (overall Cronbach’s alpha 0.793; present study Cronbach’s alpha: 0.844). In this study, the RWS was used in its Turkish version, which had previously undergone a rigorous process of cross-cultural adaptation and psychometric validation for Turkish mothers. This process included forward and backward translation, expert panel review, pre-testing, and confirmatory factor analysis, consistent with international guidelines^(12, 13, 14)^. Recent integrative reviews also confirm that cross- cultural adaptation and psychometric revalidation are essential for breastfeeding-related scales to ensure measurement equivalence and contextual relevance in different populations^(15)^.

#### Data Collection

Data were collected through face-to-face interviews in an interview room at the FHC after obtaining informed consent from the mothers. Mothers completed the forms themselves (self-report). The data collection lasted approximately 15–20 min for each participant.

### Data Analysis

Data were analyzed using IBM SPSS Statistics version 28. Descriptive statistics (frequencies, percentages, means, standard deviations, medians, and interquartile ranges) were used to summarize the variables. Normality of continuous data was tested using the Kolmogorov-Smirnov test. Group differences in Readiness for Weaning Scale (RWS) scores were examined using independent t-tests and one-way ANOVA. Associations between categorical variables and weaning behaviors were assessed using chi-square and Fisher’s exact tests. Variables with *p <* .20 in bivariate analyses were entered into multivariable logistic regression models using stepwise forward selection. Only variables with *p <* .05 were retained in the final models. Adjusted odds ratios (aORs) and 95% confidence intervals (CIs) were reported. Model fit was evaluated using the Omnibus Test of Model Coefficients, Nagelkerke’s R^2^, and the Hosmer-Lemeshow test. Sensitivity and specificity were calculated to assess classification performance. Statistical significance was set at *p <* .05.

### Ethical Considerations

This study was approved by the Human Research Health and Sports Sciences Ethics Committee of Erzincan Binali Yıldırım University (Meeting Date: 29 August 2024, Protocol No: 07-08/09). Permission to conduct the study was obtained from the Provincial Directorate of Health in the province where the study was conducted (Document ID: E-15872173-604.01-257557015). The responsible author provided permission to use the scale. Informed consent was obtained from all the mothers, and the study was conducted following the Declaration of Helsinki.

## RESULTS

The study included 202 mothers, with a mean age of 30.3 ± 5.7 years. Most participants were unemployed (72.3%) and nearly half had university-level education or higher (46.5%). Their spouses were, on average, 33.5 ± 5.9 years old, with most employed (95.0%) and over half holding a university degree (56.4%). The median age of infants was 20 months [IQR: 18–24], and most families reported a middle-income level (57.9%). Regarding infant feeding, 50.5% of mothers introduced complementary feeding at six months, 33.2% after six months, and 16.3% before six months. Detailed characteristics are summarized in Table 1.

**Table 1:** Socio-demographic characteristics of participants – Erzincan, Türkiye, 2024.

Characteristics	Overall population (N = 202)
Age (years)	30.25 ± 5.68
Spouse Age (years)	33.51 ± 5.89
Baby Age (months)	20 [18–24]
Number of Living Children	2.05 ± 1.21
Education Level	
Primary School	23 (11.4)
Middle School	34 (16.8)
High School	51 (25.2)
University and Above	94 (46.5)
Employment Status	
Employed	56 (27.7)
Unemployed	146 (72.3)
Spouse’s Education Level	
Primary School	11 (5.4)
Middle School	22 (10.9)
High School	55 (27.2)
University and Above	114 (56.4)
Spouse’s Employment Status	
Employed	192 (95.0)
Unemployed	10 (5.0)
Income Level	
Low	10 (5.0)
Medium	117 (57.9)
High	75 (37.1)
Last Birth Type	
Cesarean Birth	97 (48.0)
Vaginal Birth	105 (52.0)
Baby’s Gender	
Girl	88 (43.6)
Boy	114 (56.4)
Complementary Feeding Start	
Before 6 months	33 (16.3)
At 6 months	102 (50.5)
After 6 months	67 (33.2)

**Note:** Values are presented as mean ± SD (continuous variables), median [IQR] (non-normally distributed variables), or n (%) (categorical variables).

The mean total Readiness for Weaning Scale (RWS) score among participants was 63.68 ± 7.30 (range: 40–84). For the subscales, mean scores were as follows: Weaning Intention 19.73 ± 4.67 (range: 6–30), Infant Readiness 15.11 ± 2.80 (range: 9–22), Weaning Technique 18.39 ± 3.35 (range: 7–25), and Maternal Readiness 10.46 ± 2.55 (range: 3–15).

Table 2 summarizes the Readiness for Weaning Scale (RWS) scores across maternal socio-demographic and behavioral characteristics. Mothers who considered weaning, did not smoke, had planned pregnancies, received structured breastfeeding education, practiced regular breastfeeding, and perceived their milk supply as sufficient had significantly higher RWS scores (all *p* < 0.05). Weaning readiness was significantly associated with education level (*p* = 0.006), with university-educated mothers showing the highest scores. Income level was not significantly associated with RWS scores (*p* = 0.463). Breastfeeding support sources also significantly influenced readiness (*p* = 0.004); mothers who received professional support (nurses, midwives, or doctors) or lacked any support reported higher readiness, whereas those whose primary support came from mothers-in law or siblings had lower readiness scores.

**Table 2:** Comparison of socio-demographic characteristics with Readiness for Weaning Scale (RWS) scores (N = 202) – Erzincan, Türkiye, 2024.

Demographic characteristics	Readiness for weaning scale	
	(Mean ± SD)	Test value (t/F), p-value
Weaning Considered		t = 4.016, p = .000**
Yes (n = 92)	66.12 ± 7.10	
No (n = 110)	61.75 ± 6.85	
Smoking Status		t = *-*2.143, p = .037*
Yes (n = 11)	59.20 ± 5.10	
No (n = 191)	64.10 ± 7.30	
Planned Pregnancy		t = 1.102, p = .045*
Yes (n = 132)	64.90 ± 7.00	
No (n = 16)	61.50 ± 6.80	
Breastfeeding Education Received		t = 2.154, p = .032*
Yes (n = 87)	64.80 ± 7.00	
No (n = 115)	61.80 ± 6.90	
Regular Breastfeeding Practice		t = *-*2.486, p = .015*
Yes (n = 165)	66.48 ± 7.34	
No (n = 37)	63.45 ± 7.18	
Milk Sufficiency		t = *-*2.112, p = .036*
Yes (n = 139)	64.33 ± 7.22	
No (n = 63)	61.98 ± 7.47	
Education Level		F = 4.327, p = .006**
Primary School (n = 23)	64.15 ± 7.80	
Middle School (n = 34)	60.85 ± 7.72	
High School (n = 51)	62.55 ± 5.78	
University and Above (n = 94)	65.30 ± 7.40	
Income Level		F = 0.772, p = .463
Poor (n = 10)	61.25 ± 6.12	
Average (n = 117)	63.95 ± 7.34	
Good (n = 75)	64.25 ± 7.18	
Breastfeeding Main Support		F = 3.576, p = .004**
Spouse (n = 58)	64.85 ± 7.48	
Mother (n = 81)	63.41 ± 6.79	
Mother-in-law (n = 15)	58.70 ± 6.88	
Sibling (n = 6)	57.55 ± 5.07	
Nurse/Midwife/Doctor (n = 29)	65.02 ± 7.14	
No one (n = 8)	69.95 ± 6.64	
Friend (n = 5)	66.85 ± 9.58	

**Note:** t = independent t-test, F = one-way ANOVA, *p < .05 is considered statis­tically significant

**p < .01 is highly significant.

Table 3 presents associations between socio-demographic characteristics and maternal decisions related to weaning. Mothers with planned pregnancies, formal breastfeeding education, perception of adequate milk supply, and higher education levels were significantly more likely to consider weaning (all *p <* 0.05). Planned pregnancy and lower education levels were also strongly associated with the use of traditional weaning methods (*p* ≤ 0.001). Breastfeeding education showed a marginal trend toward less reliance on traditional methods, though this was not statistically significant (*p* = 0.078). Neither breastfeeding support nor perceived milk sufficiency significantly predicted reliance on traditional methods, and income level was not significantly associated with either weaning consideration or traditional methods (all *p* > 0.05).

**Table 3:** Associations between socio-demographic factors and weaning practices: A chi-square and fisher’s exact test analysis – Erzincan, Türkiye, 2024.

Characteristic	Weaning considered			Traditional weaning use		
	Yes	No	p-value	Yes	No	p-value
Planned Pregnancy						
Yes	70 (53.0)	62 (47.0)	.048*	17 (12.9)	115 (87.1)	.001**
No	8 (50.0)	8 (50.0)		3 (18.8)	13 (81.3)	
Breastfeeding Education Received						
Yes	47 (54.0)	40 (46.0)	.045*	10 (11.5)	77 (88.5)	.078
No	45 (39.1)	70 (60.9)		28 (24.3)	87 (75.7)	
Milk Sufficiency Perception						
Yes	64 (46.0)	75 (54.0)	.029*	27 (19.4)	112 (80.6)	.647
No	28 (44.4)	35 (55.6)		14 (22.2)	49 (77.8)	
Breastfeeding Main Support						
Spouse	21 (36.2)	37 (63.8)		8 (14.0)	50 (86.0)	
Mother	42 (52.0)	39 (48.0)		14 (17.3)	67 (82.7)	
Mother-in-law	6 (40.0)	9 (60.0)	.541	3 (20.0)	12 (80.0)	.083
Sibling	3 (50.0)	3 (50.0)		2 (33.3)	4 (66.7)	
Nurse/Midwife/Doctor	21 (65.0)	11 (35.0)		6 (20.7)	23 (79.3)	
No one	3 (37.5)	5 (62.5)		5 (62.5)	3 (37.5)	
Friend	2 (40.0)	3 (60.0)		1 (20.0)	4 (80.0)	
Education Level						
Primary School	12 (52.2)	11 (47.8)		12 (52.2)	11 (47.8)	
Middle School	5 (14.7)	29 (85.3)	.006**	5 (14.7)	29 (85.3)	.001**
High School	7 (13.7)	44 (86.3)		7 (13.7)	44 (86.3)	
University and Above	17 (18.1)	77 (81.9)		17 (18.1)	77 (81.9)	

**Note**: Values are expressed as count and percentage. *p < .05 is considered statistically significant

**p < .01 is highly significant.

In the multivariable analysis (significance threshold: *p* < 0.05), mothers who perceived their milk supply as insufficient were approximately twice as likely to consider weaning. No other socio-demographic characteristics—including education level, planned pregnancy, breastfeeding education, or breastfeeding support—were significantly associated with weaning consideration. For traditional weaning method use, mothers with a middle school education were significantly less likely to use traditional methods compared to those with primary school education. Other factors, such as planned pregnancy, breastfeeding education, milk sufficiency perception, and breastfeeding support, were not significantly associated. While the regression models identified statistically significant predictors, the overall explanatory power was modest (Nagelkerke *R* ^2^ = 0.081 and 0.185, respectively), suggesting the influence of other unmeasured factors (Table 4).

**Table 4:** Multivariable logistic regression analysis of features affecting weaning consideration and traditional weaning method use (N = 202) – Erzincan, Türkiye, 2024.

Characteristics	Odds ratio	% 95 CI		Wald	p	Odds ratio	% 95 CI		Wald	p
		Lower	Upper				Lower	Upper
Planned Pregnancy										
Yes (Ref)	1.000	–	–	–	–	1.000	–	–	–	–
No, unwanted	0.588	0.288	1.202	2.115	.146	0.381	0.135	1.077	3.313	.069
Not planned, but wanted	0.687	0.209	2.258	0.383	.536	0.324	0.072	1.452	2.168	.141
Breastfeeding Education Received										
Yes (Ref)	1.000	–	–	–	–	1.000	–	–	–	–
No	0.793	0.417	1.506	0.503	.478	1.844	0.791	4.296	2.010	.156
Milk Sufficiency Perception										
Yes (Ref)	1.000	–	–	–	–	1.000	–	–	–	–
No	1.918	1.003	3.670	3.875	.041*	0.936	0.406	2.155	0.024	.876
Education Level										
Primary School (Ref)	1.000	–	–	–	–	1.000	–	–	–	–
Middle School	0.881	0.328	2.365	0.063	.802	0.248	0.084	0.731	6.392	.030*
High School	0.827	0.348	1.966	0.185	.667	1.388	0.426	4.521	0.296	.587
University and Above	0.672	0.325	1.387	1.158	.282	1.423	0.522	3.880	0.475	.491
Breastfeeding Main Support										
Spouse (Ref)	1.000	–	–	–	–	1.000	–	–	–	–
Mother	1.565	0.219	11.180	0.200	.655	2.228	0.188	26.403	0.403	.525
Mother-in-law	1.003	0.141	7.127	0.000	.998	3.025	0.251	36.507	0.759	.384
Sibling	1.107	0.127	9.655	0.008	.927	2.457	0.163	36.930	0.423	.516
Nurse/Midwife/Doctor	0.746	0.062	8.939	0.053	.817	0.839	0.040	17.416	0.013	.910
No one	0.647	0.086	4.848	0.180	.672	1.837	0.150	22.467	0.227	.634
Friend	1.531	0.143	16.414	0.124	.725	0.326	0.019	5.465	0.607	.436
Constant	1.323	–	–	0.082	.775	3.587	–	–	1.088	.297

**Note:** Odds Ratio = Exp(B); CI = Confidence Interval; *p < .05 indicates statistical significance. Model fit for Weaning Considered: Omnibus *χ* ^2^(13) = 12.602, *p* = .479; Nagelkerke R^2^ = .081; Correct classification = 61.9%; Hosmer–Lemeshow goodness-of-fit: *χ* ^2^(8) = 11.084, *p* = .197. Model fit for Traditional Weaning Use: Omnibus *χ* ^2^(13) = 25.229, *p* = .022; Nagelkerke R^2^ = .185; Correct classification = 82.7%; Hosmer–Lemeshow goodness-of-fit: *χ* ^2^(8) = 14.255, *p* = .075.

## DISCUSSION

Breastfeeding is widely recognized for its substantial health benefits, yet the decision to initiate weaning remains a complex process shaped by a multitude of socio-demographic and behavioral factors. Previous research has emphasized the pivotal roles of maternal education, employment status, and social support networks in influencing both breastfeeding duration and readiness for weaning^(16)^. However, much of the literature has concentrated on breastfeeding initiation and continuation, with less focus on the factors determining mothers’ readiness to transition from breastfeeding^(17)^. While some studies have examined the influence of maternal behaviors and socio-economic variables on early weaning^(18)^, the interplay between these factors and readiness for weaning remains poorly understood. This study addressed this gap by evaluating maternal readiness for weaning and identifying its socio-demographic and behavioral determinants.

The mean Readiness for Weaning Scale (RWS) score in this study was 63.68 ± 7.30, suggesting a moderate level of readiness to wean. Subscale scores were highest for Weaning Intention (19.73 ± 4.67) and Weaning Technique (18.39 ± 3.35), and lower for Infant Readiness (15.11 ± 2.80) and Maternal Readiness (10.46 ± 2.55). This pattern, in which emotional and behavioral readiness exceed cognitive understanding, aligns with findings from other maternal assessment tools. For example, Brandão et al.^(19)^ found higher emotional confidence than informational scores in the Breastfeeding Self-Efficacy Scale^(19)^. Similarly, Peñacoba-Puente et al.^(20)^ observed stronger maternal motivation scores compared to knowledge domains in the Breastfeeding Motivation Scale^(20)^. The Turkish version of the RWS demonstrated good internal consistency in our sample (Cronbach’s *α* = 0.84), consistent with the original scale development by Türkmen et al.^(11)^. Nonetheless, further construct and cross-cultural validation are warranted, as instrument performance may vary across cultural and contextual boundaries. Wood et al.^(21)^ emphasized that even reliable scales must be reassessed across populations to ensure construct equivalence and cultural fit^(21)^. Similarly, Abbass-Dick et al.^(22)^ demonstrated the need for predictive validity and external consistency checks when applying new instruments in diverse settings^(22)^. These considerations are essential to ensure that scores reflect meaningful differences in maternal readiness and can reliably guide clinical or educational interventions in the Turkish context.

Multivariate logistic regression, using a stepwise method, included predictors with bivariate significance at *p* < 0.20. The final model identified perception of milk sufficiency, maternal education, breastfeeding education, and planned pregnancy as significant predictors of weaning consideration (all *p* < 0.05). These findings align with Ayesha et al.^(23)^, who reported that mothers confident in their milk supply were more likely to transition from breastfeeding intentionally (aOR = 2.14, 95% CI: 1.17–3.91)^(23)^. Maternal education has also been associated with higher feeding autonomy and evidence-based practices, as confirmed by Kopp et al.^(24)^, who found that higher education and planned pregnancy predicted structured infant feeding decisions (aOR = 1.92, 95% CI: 1.14–3.01)^(24)^. While breastfeeding education was significant in our model, breastfeeding support did not predict weaning consideration, consistent with Gizaw et al.^(25)^, who noted that support not tailored to sociocultural contexts often lacks behavioral impact^(25)^. Sarpkaya Güder and İnce^(9)^ also observed that general support interventions had limited effectiveness unless culturally aligned, particularly among mothers with lower education^(9)^. Our model explained 18.5% of the variance (Nagelkerke *R* ^2^ = 0.185) and correctly classified 82.7% of cases. Comparable models have been validated elsewhere: Yakovlev et al.^(26)^ reported that internet-based breastfeeding education reduced weaning risk (OR = 0.65, 95% CI: 0.43–0.96)^(26)^, and Rassie et al.^(27)^ demonstrated strong model fit for maternal metabolic and social predictors of breastfeeding (GEE ORs: 0.54–1.73)^(27)^. Psychosocial dimensions are also relevant; Yeni et al.^(28)^ found maternal behavior strongly predicted infant readiness to wean (*p* = 0.002)^(28)^, while Mathews et al.^(29)^ identified emotional regulation and feeding beliefs as early predictors of weaning transitions^(29)^. Collectively, these findings reinforce the need to assess weaning within a culturally and psychosocially nuanced framework, supported by robust model diagnostics and updated evidence.

It is notable that breastfeeding support did not significantly predict weaning consideration or reliance on traditional methods, as also observed in the chi-square analyses. This may reflect the complexity of support dynamics in Turkish society, where extended family involvement can sometimes reinforce traditional practices rather than support evidence-based weaning. The high classification accuracy for traditional weaning use (82.7%) was primarily driven by a strong ability to predict non-reliance on these methods. Several methodological limitations should be considered when interpreting these findings. First, the use of self-reported questionnaires introduces the risk of recall bias and social desirability effects, which may affect the accuracy of responses. Second, although we presented key statistical outputs and model adjustment metrics, the explanatory power of the regression models was moderate, and unmeasured variables may contribute to maternal weaning decisions. Future research should seek to validate the RWS in diverse Turkish populations and other sociocultural contexts, ideally using both quantitative and qualitative methods to provide richer insights. Multicenter or longitudinal studies, as well as qualitative interviews exploring family dynamics and cultural beliefs around weaning, are recommended to address the limitations of self-report and strengthen the evidence base for policy and practice. Despite these limitations, our study contributes valuable evidence on the socio-demographic and behavioral correlates of weaning readiness in Turkish mothers, and underscores the need for culturally tailored interventions to support optimal weaning practices.

## CONCLUSION

This study revealed that maternal education, structured breastfeeding education, milk sufficiency perception, and planned pregnancy were significantly associated with greater readiness for weaning. Mothers with higher education levels and formal breastfeeding guidance demonstrated greater autonomy in feeding decisions and were less reliant on traditional weaning practices. However, the study faced several limitations. First, the Readiness for Weaning Scale (RWS) was originally developed and psychometrically validated among Turkish mothers, demonstrating good validity and reliability for use in this population. However, as with all measurement tools, additional validation in diverse Turkish regions and subgroups would further strengthen its generalizability. Second, reliance on self-reported data may introduce recall and social desirability bias. Third, although statistical models were presented, their explanatory power was moderate, and unmeasured factors likely influence maternal decisions. To build on these findings, future research should consider qualitative approaches to explore maternal perceptions of weaning readiness in depth. Additionally, multicenter studies involving diverse regions and populations are recommended to enhance generalizability. Future multicenter studies should also complete a full COSMIN-guided validation of the RWS to confirm factor structure, invariance and responsiveness in diverse Turkish settings.

## Data Availability

The dataset supporting the results of this study is not publicly available due to privacy or ethical restrictions.
