# Supplementary material for: Maternal readiness for weaning: Influencing factors and determinants
Source: Rev Esc Enferm USP. 2025 Sep 26;59:e20250163. doi: 10.1590/1980-220X-REEUSP-2025-0163en (PMC12466449; doi:10.1590/1980-220X-REEUSP-2025-0163en)
Supplement: Translation

# Translation: Preparación materna para el destete: factores influyentes y determinantes

**DOI:** 10.1590/1980-220X-REEUSP-2025-0163es

**Authors:** Kamile Akça, Songül Kekil, Meltem Özcan

**Affiliations:** 1 Gaziantep Islam Science and Technology University, Faculty of Health Sciences, Gaziantep, Türkiye.; 2 Erzincan Binali Yıldırım University, Health Services Vocational School, Erzincan, Türkiye.

**Keywords:** Lactancia Materna, Destete, Conducta Materna, Lactante, Lactancia Materna Complementaria

## Abstract

**Objetivo:**

Evaluar la preparación materna para el destete y examinar los factores sociodemográficos y conductuales que influyen en dicha decisión.

**Método:**

Estudio transversal llevado a cabo en tres Centros de Salud Familiar de una capital provincial entre octubre de 2024 y enero de 2025, con 202 madres lactantes. Los datos se recogieron mediante entrevistas presenciales utilizando un cuestionario sociodemográfico y la Escala de Preparación para el Destete validada. Los análisis estadísticos incluyeron pruebas *t*, ANOVA, chi-cuadrado y regresión logística.

**Resultados:**

Las madres con mayor nivel educativo presentaron puntuaciones significativamente más altas. La educación sobre lactancia, el embarazo planificado y la percepción de suficiencia de leche se asociaron positivamente con la preparación; el tabaquismo se asoció negativamente. La percepción de suficiencia de leche predijo la consideración del destete; menor educación se vinculó con métodos tradicionales.

**Conclusión:**

La educación materna, la preparación psicológica y el apoyo profesional son fundamentales para un destete estructurado. Se necesitan intervenciones dirigidas para mejorar la confianza materna y la toma de decisiones informadas. Limitaciones diseño transversal y datos autoinformados pueden limitar la inferencia causal e introducir sesgos de recuerdo o deseabilidad social.

## INTRODUCCIÓN

La lactancia materna está ampliamente reconocida como el método óptimo de nutrición infantil, ya que ofrece numerosos beneficios para la salud tanto de la madre como del niño. La Organización Mundial de la Salud (OMS) recomienda la lactancia materna exclusiva durante los primeros seis meses de vida, seguida de la lactancia materna continuada con alimentos complementarios hasta los dos años o más^(1)^. A pesar de estas recomendaciones, las tasas de cumplimiento a nivel mundial siguen siendo insuficientes, y muchas madres interrumpen la lactancia materna antes de lo recomendado debido a diversos factores sociodemográficos, psicológicos y culturales^(2)^. El destete precoz puede verse influido por múltiples factores, entre ellos el empleo de la madre, su nivel educativo, el apoyo familiar y la orientación sanitaria^(3)^.

La decisión de la madre sobre el destete es compleja y está determinada por factores tanto individuales como ambientales. Las investigaciones sugieren que un mayor nivel de educación materna se asocia con una mayor duración de la lactancia materna y una toma de decisiones informada sobre el destete^(4)^. Además, la experiencia previa en la lactancia materna, la educación prenatal y el apoyo social desempeñan un papel importante en la preparación de la madre para el destete^(5,6)^. Mientras que algunos estudios destacan la influencia positiva del apoyo profesional a la lactancia y la educación estructurada sobre la lactancia materna^(7)^, otros señalan las expectativas culturales y las creencias tradicionales como obstáculos para un destete oportuno y estructurado^(8)^. Por ejemplo, en algunas zonas rurales de Uganda y el norte de Chipre, las madres suelen utilizar sustancias herbales, aplicar extractos de plantas de sabor amargo en los senos o recurrir a la interrupción repentina para fomentar el destete, prácticas que son culturalmente aceptadas pero que pueden entrar en conflicto con las directrices basadas en la evidencia^(8,9)^. Estos métodos tradicionales suelen transmitirse de generación en generación y reflejan normas culturales profundamente arraigadas, especialmente entre las poblaciones con un nivel educativo formal más bajo.

A pesar de las numerosas investigaciones sobre el inicio y la duración de la lactancia materna, existe una notable brecha en la comprensión de la preparación de las madres para el destete y los factores que influyen en esta transición. Abordar esta brecha es fundamental para desarrollar intervenciones específicas que ayuden a las madres a tomar decisiones informadas y seguras sobre el destete. Por lo tanto, el objetivo de este estudio es evaluar la preparación de las madres para el destete y examinar los factores sociodemográficos y conductuales que influyen en las decisiones de destete.

## MÉTODO

### Diseño y Entorno del Estudio

Este estudio empleó un diseño transversal y utilizó cuestionarios estructurados para evaluar la preparación de las madres para el destete. El estudio se llevó a cabo en tres centros de salud familiar (CSF) situados en un centro provincial del este de Turquía.

### Población y Muestra

La población del estudio estaba formada por madres que acudieron a los centros de salud familiar (CSF) por cualquier motivo entre octubre de 2024 y enero de 2025. La muestra del estudio incluyó a madres con bebés de 18 meses o más que aún estaban amamantando en el momento de la recolección de datos. Los criterios de inclusión adicionales fueron: residencia en la provincia durante al menos seis meses, capacidad para comunicarse en turco y alfabetización adecuada para comprender y responder a los instrumentos del estudio. Los criterios de exclusión incluyeron madres con discapacidades visuales, auditivas, del habla o físicas, trastornos psiquiátricos, partos múltiples (gemelos o trillizos) o bebés con enfermedades crónicas o afecciones de salud significativas. El tamaño de la muestra para este estudio se determinó mediante un análisis de potencia a priori utilizando G*Power 3.1.9.7, teniendo en cuenta el enfoque estadístico primario del análisis de regresión logística. Dado que ningún estudio anterior había utilizado la Escala de Preparación para el Destete (Readiness for Weaning Scale – RWS), no se disponía de estimaciones del tamaño del efecto. Por lo tanto, basamos nuestro cálculo en un nivel de significación de α = 0,05, una potencia de 0,80 y un tamaño del efecto medio estimado (f^2^ de Cohen = 0,15), siguiendo las recomendaciones estándar en la investigación en ciencias del comportamiento y de la salud^(10)^. El análisis de potencia se realizó para detectar una relación significativa entre los factores sociodemográficos y la preparación materna para el destete. El análisis indicó que se necesitaría un mínimo de 178 participantes para alcanzar una potencia estadística suficiente. Teniendo en cuenta este requisito, se reclutó a un total de 202 madres para garantizar un tamaño de muestra que cumpliera el umbral de potencia y permitiera una posible pérdida de datos. Además, el tamaño de la muestra cumplía el criterio de al menos 10-20 eventos por variable predictiva (events for predictive variable – EPV) necesario para un análisis de regresión logística estable.

### Medidas

#### Características Sociodemográficas

Se utilizó el cuestionario de características socio­demográficas para recolectar información detallada sobre las características maternas e infantiles relevantes para las prácticas de lactancia materna y destete. A los efectos de este estudio, también se evaluaron los métodos tradicionales de destete. Los métodos tradicionales de destete se definen como prácticas culturalmente arraigadas y comúnmente utilizadas en el contexto local. Entre ellos se incluyen el cese brusco de la lactancia materna, la aplicación de sustancias herbales o alimentos de sabor amargo en los senos para disuadir de la lactancia, y la dependencia de las tradiciones familiares o los consejos de familiares mayores (como las abuelas o las suegras) en lugar de las recomendaciones profesionales. Estos métodos se diferencian de los enfoques de destete gradual, estructurado o guiado por profesionales.

#### Escala de Preparación para el Destete (RWS)

La Escala de preparación para el destete (RWS) es un instrumento de autoevaluación desarrollado para evaluar la preparación de las madres para la transición de la lactancia materna^(11)^. La RWS consta de 19 ítems distribuidos en cuatro dimensiones: intención de destete, preparación del bebé, técnica de destete y preparación de la madre. Cada ítem se puntúa en una escala de Likert de 5 puntos, que va de 1 (totalmente en desacuerdo) a 5 (totalmente de acuerdo), y las puntuaciones más altas indican una mayor preparación de la madre para el destete. La puntuación total de la RWS se obtiene sumando todas las respuestas de los ítems, lo que da un rango de 19 a 95. Dado que no existen valores de corte universales, interpretamos las puntuaciones que se situaban al menos una desviación estándar por encima de la media de la muestra como «mayor preparación» y las que se situaban al menos una desviación estándar por debajo de la media como «menor preparación», siguiendo la práctica psicométrica actual. En este estudio, la escala se utilizó en su forma original validada en turco, desarrollada y validada psicométricamente en una población turca^(11)^. El proceso de validación incluyó la evaluación de la validez del constructo y la consistencia interna (alfa de Cronbach global 0,793; alfa de Cronbach del presente estudio: 0,844). En este estudio, se utilizó la RWS en su versión turca, que había sido sometida previamente a un riguroso proceso de adaptación intercultural y validación psicométrica para madres turcas. Este proceso incluyó traducción directa e inversa, revisión por un panel de expertos, pruebas previas y análisis factorial confirmatorio, de acuerdo con las directrices internacionales^(12, 13, 14)^. Revisiones integradoras recientes también confirman que la adaptación intercultural y la revalidación psicométrica son esenciales para las escalas relacionadas con la lactancia materna a fin de garantizar la equivalencia de la medición y la relevancia contextual en diferentes poblaciones^(15)^.

### Recolección De Datos

Los datos se recolectaron mediante entrevistas presenciales en una sala de entrevistas del CSF, tras obtener el consentimiento informado de las madres. Las madres completaron los formularios ellas mismas (autoinforme). La recolección de datos duró aproximadamente entre 15 y 20 minutos para cada participante.

### Análisis de Datos

Los datos se analizaron utilizando IBM SPSS Statistics versión 28. Se utilizaron estadísticas descriptivas (frecuencias, porcentajes, medias, desviaciones estándar, medianas y rangos intercuartílicos) para resumir las variables. La normalidad de los datos continuos se comprobó mediante la prueba de Kolmogorov-Smirnov. Las diferencias entre los grupos en las puntuaciones de la Escala de Preparación para el Destete (RWS) se examinaron mediante pruebas t independientes y ANOVA unidireccional. Las asociaciones entre las variables categóricas y los comportamientos de destete se evaluaron mediante pruebas de chi cuadrado y exactas de Fisher. Las variables con *p <* 0,20 en los análisis bivariados se introdujeron en modelos de regresión logística multivariable utilizando selección escalonada hacia adelante. Solo se mantuvieron en los modelos finales las variables con *p <* 0,05. Se informaron las odds ratio ajustadas (aOR) y los intervalos de confianza (IC) del 95%. El ajuste del modelo se evaluó mediante la prueba Omnibus de coeficientes del modelo, el R^2^ de Nagelkerke y la prueba de Hosmer-Lemeshow. Se calcularon la sensibilidad y la especificidad para evaluar el rendimiento de la clasificación. La significación estadística se fijó en *p* < 0,05.

### Consideraciones Éticas

Este estudio fue aprobado por el Comité de Ética en Investigación con Seres Humanos y Ciencias de la Salud y el Deporte de la Universidad Erzincan Binali Yıldırım (fecha de la reunión: 29 Agosto 2024, protocolo n.º: 07-08/09). Se obtuvo permiso para realizar el estudio de la Dirección Provincial de Salud de la provincia donde se llevó a cabo (Documento ID: E-15872173-604.01-257557015). El autor responsable dio permiso para utilizar la escala. Se obtuvo el consentimiento informado de todas las madres y el estudio se llevó a cabo siguiendo la Declaración de Helsinki.

## RESULTADOS

En el estudio participaron 202 madres, con una edad media de 30,3 ± 5,7 años. La mayoría de las participantes estaban desempleadas (72,3%) y casi la mitad tenían estudios universitarios o superiores (46,5%). Sus cónyuges tenían una edad media de 33,5 ± 5,9 años, la mayoría estaban empleados (95,0%) y más de la mitad tenían un título universitario (56,4%). La mediana de edad de los bebés era de 20 meses [IQR: 18–24], y la mayoría de las familias declararon tener un nivel de ingresos medio (57,9%). En cuanto a la alimentación de los bebés, el 50,5% de las madres introdujeron alimentos complementarios a los seis meses, el 33,2% después de los seis meses y el 16,3% antes de los seis meses. Las características detalladas se resumen en la Tabla 1.

**Tabla 1:** Características sociodemográficas de las participantes – Erzincan, Turquía, 2024.

Características	Población total (N = 202)
Edad (años)	30,25 ± 5,68
Edad del cónyuge (años)	33,51 ± 5,89
Edad del bebé (meses)	20 [18–24]
Número de hijos vivos	2,05 ± 1,21
Nivel educativo	
Primaria	23 (11,4)
Secundaria	34 (16,8)
Secundaria	51 (25,2)
Universidad y superior	94 (46,5)
Situación laboral	
Empleado	56 (27,7)
Sin empleo	146 (72,3)
Nivel educativo del cónyuge	
Primaria	11 (5,4)
Secundaria	22 (10,9)
Secundaria	55 (27,2)
Universitaria y superior	114 (56,4)
Situación laboral del cónyuge	
Empleado	192 (95,0)
Desempleado	10 (5,0)
Nivel de ingresos	
Bajo	10 (5,0)
Medio	117 (57,9)
Alta	75 (37,1)
Tipo de último parto	
Cesárea	97 (48,0)
Parto vaginal	105 (52,0)
Sexo del bebé	
Niña	88 (43,6)
Niño	114 (56,4)
Inicio de la alimentación complementaria	
Antes de los 6 meses	33 (16,3)
A los 6 meses	102 (50,5)
Después de los 6 meses	67 (33,2)

**Nota:** Los valores se presentan como media ± Desvío Estándar (variables continuas), mediana [IQR] (variables con distribución no normal) o n (%) (variables categóricas).

La puntuación media total en la Escala de Preparación para el Destete (RWS) entre los participantes fue de 63,68 ± 7,30 (rango: 40–84). En cuanto a las subescalas, las puntuaciones medias fueron las siguientes: intención de destete, 19,73 ± 4,67 (rango: 6–30); preparación del lactante, 15,11 ± 2,80 (rango: 9–22); Técnica de destete 18,39 ± 3,35 (rango: 7–25) y Preparación materna 10,46 ± 2,55 (rango: 3–15).

La Tabla 2 resume las puntuaciones de la Escala de Preparación para el Destete (RWS) según las características sociodemográficas y conductuales de las madres. Las madres que consideraban el destete, no fumaban, habían planeado el embarazo, habían recibido educación estructurada sobre lactancia materna, practicaban la lactancia materna regularmente y percibían que su producción de leche era suficiente obtuvieron puntuaciones significativamente más altas en la RWS (todas *p* < 0,05). La preparación para el destete se asoció significativamente con el nivel educativo (p = 0,006), y las madres con estudios universitarios obtuvieron las puntuaciones más altas. El nivel de ingresos no se asoció significativamente con las puntuaciones de la RWS (p = 0,463). Las fuentes de apoyo a la lactancia materna también influyeron significativamente en la preparación (p = 0,004); las madres que recibieron apoyo profesional (enfermeras, comadronas o médicos) o que carecían de cualquier tipo de apoyo declararon una mayor preparación, mientras que aquellas cuyo apoyo principal procedía de sus suegras o hermanos obtuvieron puntuaciones más bajas en la escala de preparación.

**Tabla 2:** Comparación de las características sociodemográficas con las puntuaciones de la Escala de Preparación para el Destete (RWS) (N = 202) – Erzincan, Turquía, 2024.

Características demográficas	Escala de preparación para el destete	
	(Media ± Desvío Estándar)	Valor de la prueba (t/F), valor p
Se considera el destete		t = 4,016, p = 0,000**
Sí (n = 92)	66,12 ± 7,10	
No (n = 110)	61,75 ± 6,85	
Estado de tabaquismo		t = -2,143, p = 0,037*
Sí (n = 11)	59,20 ± 5,10	
No (n = 191)	64,10 ± 7,30	
Embarazo planificado		t = 1,102, p = 0,045*
Sí (n = 132)	64,90 ± 7,00	
No (n = 16)	61,50 ± 6,80	
Educación sobre lactancia materna recibida		t = 2,154, p = 0,032*
Sí (n = 87)	64,80 ± 7,00	
No (n = 115)	61,80 ± 6,90	
Práctica regular de lactancia materna		t = -2,486, p = 0,015*
Sí (n = 165)	66,48 ± 7,34	
No (n = 37)	63,45 ± 7,18	
Suficiencia láctea		t = -2,112, p = 0,036*
Sí (n = 139)	64,33 ± 7,22	
No (n = 63)	61,98 ± 7,47	
Nivel educativo		F = 4,327, p = 0,006**
Escuela primaria (n = 23)	64,15 ± 7,80	
Secundaria (n = 34)	60,85 ± 7,72	
Secundaria (n = 51)	62,55 ± 5,78	
Universidad y superior (n = 94)	65,30 ± 7,40	
Nivel de ingresos		F = 0,772, p = 0,463
Pobre (n = 10)	61,25 ± 6,12	
Promedio (n = 117)	63,95 ± 7,34	
Bueno (n = 75)	64,25 ± 7,18	
Lactancia materna como principal fuente de apoyo		F = 3,576, p = 0,004**
Cónyuge (n = 58)	64,85 ± 7,48	
Madre (n = 81)	63,41 ± 6,79	
Suegra (n = 15)	58,70 ± 6,88	
Hermano (n = 6)	57,55 ± 5,07	
Enfermera/comadrona/médico (n = 29)	65,02 ± 7,14	
Nadie (n = 8)	69,95 ± 6,64	
Amiga (n = 5)	66,85 ± 9,58	

**Nota:** t = prueba t independiente, F = ANOVA unidireccional, *p < 0,05 se con­sidera estadísticamente significativo

**p < 0,01 es muy significativo.

La Tabla 3 presenta las asociaciones entre las características sociodemográficas y las decisiones maternas relacionadas con el destete. Las madres con embarazos planificados, educación formal sobre lactancia materna, percepción de una producción adecuada de leche y niveles de educación más altos eran significativamente más propensas a considerar el destete (todas p *<* o 0,05). El embarazo planificado y los niveles de educación más bajos también se asociaron fuertemente con el uso de métodos tradicionales de destete (*p* ≤ 0,001). La educación sobre lactancia materna mostró una tendencia marginal hacia una menor dependencia de los métodos tradicionales, aunque esto no fue estadísticamente significativo (p = 0,078). Ni el apoyo a la lactancia materna ni la percepción de suficiencia de leche predijeron significativamente la dependencia de los métodos tradicionales, y el nivel de ingresos no se asoció significativamente con la consideración del destete ni con los métodos tradicionales (todos p > 0,05).

**Tabla 3:** Asociaciones entre los factores sociodemográficos y las prácticas de destete: análisis mediante la prueba de chi cuadrado y la prueba exacta de Fisher – Erzincan, Turquía, 2024.

Característica	Consideración del destete			Uso de métodos tradicionales de destete		
	Sí	No	valor p	Sí	No	valor p
Embarazo planificado						
Sí	70 (53,0)	62 (47,0)	0,048*	17 (12,9)	115 (87,1)	0,001**
No	8 (50,0)	8 (50,0)		3 (18,8)	13 (81,3)	
Educación sobre lactancia materna recibida						
Sí	47 (54,0)	40 (46,0)	0,045	10 (11,5)	77 (88,5)	0,078
No	45 (39,1)	70 (60,9)		28 (24,3)	87 (75,7)	
Percepción de la suficiencia de leche						
Sí	64 (46,0)	75 (54,0)	0,029	27 (19,4)	112 (80,6)	0,647
No	28 (44,4)	35 (55,6)		14 (22,2)	49 (77,8)	
Lactancia materna principal				
Cónyuge	21 (36,2)	37 (63,8)		8 (14,0)	50 (86,0)	
Madre	42 (52,0)	39 (48,0)		14 (17,3)	67 (82,7)	
Suegra	6 (40,0)	9 (60,0)	0,541	3 (20,0)	12 (80,0)	0,083
Hermano	3 (50,0)	3 (50,0)		2 (33,3)	4 (66,7)	
Enfermera/partera/médico	21 (65,0)	11 (35,0)		6 (20,7)	23 (79,3)	
Nadie	3 (37,5)	5 (62,5)		5 (62,5)	3 (37,5)	
Amiga	2 (40,0)	3 (60,0)		1 (20,0)	4 (80,0)	
Nivel educativo						
Escuela primaria	12 (52,2)	11 (47,8)		12 (52,2)	11 (47,8)	
Primer ciclo secundaria	5 (14,7)	29 (85,3)	0,006**	5 (14,7)	29 (85,3)	0,001**
Segundo ciclo secundaria	7 (13,7)	44 (86,3)		7 (13,7)	44 (86,3)	
Universidad y superior	17 (18,1)	77 (81,9)		17 (18,1)	77 (81,9)	

**Nota:** Los valores se expresan en recuento y porcentaje. *Se considera estadísticamente significativo un valor de p < 0,05

** y muy significativo un valor de p < 0,01.

En el análisis multivariante (umbral de significación: *p* < 0,05), las madres que percibían que su producción de leche era insuficiente eran aproximadamente dos veces más propensas a considerar el destete. Ninguna otra característica sociodemográfica, incluyendo el nivel educativo, la planificación del embarazo, la educación sobre lactancia materna o el apoyo a la lactancia materna, se asoció significativamente con la consideración del destete. En cuanto al uso de métodos tradicionales de destete, las madres con educación secundaria eran significativamente menos propensas a utilizar métodos tradicionales en comparación con las que tenían educación primaria. Otros factores, como la planificación del embarazo, la educación sobre lactancia materna, la percepción de la suficiencia de la leche y el apoyo a la lactancia materna, no se asociaron de manera significativa. Si bien los modelos de regresión identificaron predictores estadísticamente significativos, la capacidad explicativa general fue modesta (Nagelkerke *R* ^2^ = 0,081 y 0,185, respectivamente), lo que sugiere la influencia de otros factores no medidos (Tabla 4).

**Tabla 4:** Análisis de regresión logística multivariable de las características que influyen en la consideración del destete y el uso de métodos tradicionales de destete (N = 202) – Erzincan, Turquía, 2024.

Características	Odds ratio	IC		Wald	p	Odds ratio	IC del 95		Wald	p
		Inferior	Superior				Inferior	Superior
Embarazo planificado										
Sí (Ref)	1,000	–	–	–	–	1.000	–	–	–	–
No, no deseado	0,588	0,288	1,202	2,115	0,146	0,381	0,135	1,077	3,313	0,069
No planificado, pero deseado	0,687	0,209	2,258	0,383	0,536	0,324	0,072	1,452	2,168	0,141
Educación recibida sobre lactancia materna										
Sí (Ref)	1,000	–	–	–	–	1.000	–	–	–	–
No	0,793	0,417	1,506	0,503	0,478	1,844	0,791	4,296	2,010	0,156
Percepción de la suficiencia de leche										
Sí (Ref)	1,000	–	–	–	–	1.000	–	–	–	–
No	1,918	1,003	3,670	3,875	0,041*	0,936	0,406	2,155	0,024	0,876
Educación										
Escuela primaria (Ref)	1,000	–	–	–	–	1.000	–	–	–	–
Primer ciclo secundaria	0,881	0,328	2,365	0,063	0,802	0,248	0,084	0,731	6,392	0,030*
Segundo ciclo secundaria	0,827	0,348	1,966	0,185	0,667	1,388	0,426	4,521	0,296	0,587
Universitaria y superior	0,672	0,325	1,387	1,158	0,282	1,423	0,522	3,880	0,475	0,491
Lactancia materna Apoyo principal										
Cónyuge (Ref)	1,000	–	–	–	–	1.000	–	–	–	–
Madre	1,565	0,219	11,180	0,200	0,655	2,228	0,188	26,403	0,403	.525
Suegra	1,003	0,141	7,127	0,000	0,998	3,025	0,251	36,507	0,759	0,384
Hermanos	1,107	0,127	9,655	0,008	0,927	2,457	0,163	36,930	0,423	0,516
Enfermera/comadrona/médico	0,746	0,062	8,939	0,053	0,817	0,839	0,040	17,416	0,013	0,910
Nadie	0,647	0,086	4,848	0,180	0,672	1,837	0,150	22,467	0,227	0,634
Amiga	1,531	0,143	16,414	0,124	0,725	0,326	0,019	5,465	0,607	0,436
Constante	1,323	–	–	0,082	0,775	3,587	–	–	1,088	0,297

**Nota:** Odds Ratio = Exp(B); IC = Intervalo de confianza; *p < 0,05 indica significación estadística. Ajuste del modelo para el destete considerado: χ^2^ global (13) = 12,602, *p* = 0,479; R^2^ de Nagelkerke = 0,081; clasificación correcta = 61,9%; bondad del ajuste de Hosmer-Lemeshow: χ^2^ (8) = 11,084, *p* = 0,197. Ajuste del modelo para el uso tradicional del destete: χ^2^ global (13) = 25,229, *p* = 0,022; R^2^ de Nagelkerke = 0,185; clasificación correcta = 82,7%; bondad del ajuste de Hosmer-Lemeshow: χ^2^ (8) = 14,255, *p* = 0,075.

## DISCUSIÓN

La lactancia materna es ampliamente reconocida por sus importantes beneficios para la salud, pero la decisión de iniciar el destete sigue siendo un proceso complejo en el que influyen múltiples factores sociodemográficos y conductuales. Investigaciones anteriores han destacado el papel fundamental de la educación materna, la situación laboral y las redes de apoyo social en la duración de la lactancia materna y la preparación para el destete^(16)^. Sin embargo, gran parte de la bibliografía se ha centrado en el inicio y la continuación de la lactancia materna, prestando menos atención a los factores que determinan la disposición de las madres para pasar de la lactancia materna^(17)^. Si bien algunos estudios han examinado la influencia de los comportamientos maternos y las variables socioeconómicas en el destete precoz^(18)^, la interacción entre estos factores y la disposición para el destete sigue sin conocerse bien. El presente estudio aborda esta laguna evaluando la disposición de las madres para el destete e identificando sus determinantes sociodemográficos y conductuales.

La puntuación media de la Escala de Preparación para el Destete (RWS) en este estudio fue de 63,68 ± 7,30, lo que sugiere un nivel moderado de preparación para el destete. Las puntuaciones más altas de la subescala se obtuvieron en la intención de destete (19,73 ± 4,67) y la técnica de destete (18,39 ± 3,35), y las más bajas en la preparación del lactante (15,11 ± 2,80) y la preparación materna (10,46 ± 2,55). Este patrón, en el que la preparación emocional y conductual supera la comprensión cognitiva, concuerda con los resultados de otras herramientas de evaluación materna. Por ejemplo, Brandão et al.^(19)^ encontraron una mayor confianza emocional que puntuaciones informativas en la Escala de Autoeficacia en la Lactancia Materna^(19)^. De manera similar, Peñacoba-Puente et al.^(20)^ observaron puntuaciones más altas en la motivación materna en comparación con los dominios del conocimiento en la Escala de Motivación para la Lactancia Materna^(20)^. La versión turca de la RWS demostró una buena consistencia interna en nuestra muestra (α de Cronbach = 0,84), en consonancia con la escala original desarrollada por Türkmen et al.^(11)^. No obstante, se justifica una mayor validación constructiva e intercultural, ya que el rendimiento del instrumento puede variar según las fronteras culturales y contextuales. Wood et al.^(21)^ hicieron hincapié en que incluso las escalas fiables deben reevaluarse en diferentes poblaciones para garantizar la equivalencia del constructo y la adecuación cultural^(21)^. De manera similar, Abbass-Dick et al.^(22)^ demostraron la necesidad de comprobar la validez predictiva y la consistencia externa al aplicar nuevos instrumentos en entornos diversos^(22)^. Estas consideraciones son esenciales para garantizar que las puntuaciones reflejen diferencias significativas en la preparación materna y puedan orientar de manera fiable las intervenciones clínicas o educativas en el contexto turco.

La regresión logística multivariante, utilizando un método por pasos, incluyó predictores con significación bivariada con *p* < 0,20. El modelo final identificó la percepción de la suficiencia de la leche, la educación materna, la educación sobre la lactancia materna y el embarazo planificado como predictores significativos de la consideración del destete (todos con *p* < 0,05). Estos hallazgos coinciden con los de Ayesha et al.^(23)^, quienes informaron que las madres que confiaban en su producción de leche eran más propensas a dejar de amamantar de forma intencionada (aOR = 2,14, IC del 95%: 1,17–3,91)^(23)^. La educación materna también se ha asociado con una mayor autonomía en la alimentación y prácticas basadas en la evidencia, como confirmaron Kopp et al.^(24)^, quienes encontraron que una mayor educación y un embarazo planificado predecían decisiones estructuradas sobre la alimentación infantil (aOR = 1,92, IC del 95%: 1,14–3,01)^(24)^. Si bien la educación sobre lactancia materna fue significativa en nuestro modelo, el apoyo a la lactancia materna no predijo la consideración del destete, en consonancia con Gizaw et al.^(25)^, quienes señalaron que el apoyo que no se adapta a los contextos socioculturales a menudo carece de impacto en el comportamiento^(25)^. Sarpkaya Güder e İnce^(9)^ también observaron que las intervenciones de apoyo general tenían una eficacia limitada a menos que se adaptaran a la cultura, especialmente entre las madres con un nivel educativo más bajo^(9)^. Nuestro modelo explicó el 18,5% de la varianza (*R* ^2^ de Nagelkerke = 0,185) y clasificó correctamente el 82,7% de los casos. Se han validado modelos comparables en otros lugares: Yakovlev et al.^(26)^ informaron de que la educación sobre lactancia materna a través de Internet reducía el riesgo de destete (OR = 0,65, IC del 95%: 0,43–0,96)^(26)^, y Rassie et al.^(27)^ demostraron un fuerte ajuste del modelo para los predictores metabólicos y sociales de la lactancia materna (GEE OR: 0,54–1,73)^(27)^. Las dimensiones psicosociales también son relevantes; Yeni et al.^(28)^ encontraron que el comportamiento materno era un fuerte predictor de la disposición del lactante al destete (*p* = 0,002)^(28)^, mientras que Mathews et al.^(29)^ identificaron la regulación emocional y las creencias sobre la alimentación como predictores tempranos de las transiciones al destete^(29)^. En conjunto, estos hallazgos refuerzan la necesidad de evaluar el destete dentro de un marco cultural y psicosocial matizado, respaldado por diagnósticos sólidos y evidencia actualizada.

Cabe destacar que el apoyo a la lactancia materna no predijo significativamente la consideración del destete ni la dependencia de métodos tradicionales, como también se observó en los análisis de chi cuadrado. Esto puede reflejar la complejidad de la dinámica del apoyo en la sociedad turca, donde la participación de la familia extensa a veces puede reforzar las prácticas tradicionales en lugar de apoyar el destete basado en la evidencia. La alta precisión de la clasificación del uso tradicional del destete (82,7%) se debió principalmente a la gran capacidad de predecir la no dependencia de estos métodos. A la hora de interpretar estos hallazgos, deben tenerse en cuenta varias limitaciones metodológicas. En primer lugar, el uso de cuestionarios autoinformados introduce el riesgo de sesgo de recuerdo y efectos de deseabilidad social, que pueden afectar a la precisión de las respuestas. En segundo lugar, aunque presentamos resultados estadísticos clave y métricas de ajuste del modelo, la capacidad explicativa de los modelos de regresión fue moderada, y es posible que haya variables no medidas que contribuyan a las decisiones de las madres sobre el destete. Las investigaciones futuras deberían tratar de validar el RWS en diversas poblaciones turcas y otros contextos socioculturales, idealmente utilizando métodos cuantitativos y cualitativos para obtener una visión más rica. Se recomiendan estudios multicéntricos o longitudinales, así como entrevistas cualitativas que exploren la dinámica familiar y las creencias culturales en torno al destete, para abordar las limitaciones de los autoinformes y reforzar la base empírica para las políticas y la práctica. A pesar de estas limitaciones, nuestro estudio aporta datos valiosos sobre los correlatos sociodemográficos y conductuales de la preparación para el destete en las madres turcas, y subraya la necesidad de intervenciones adaptadas a la cultura para apoyar prácticas óptimas de destete.

## CONCLUSIÓN

Este estudio reveló que la educación materna, la educación estructurada sobre lactancia materna, la percepción de la suficiencia de la leche y los embarazos planificados se asociaban significativamente con una mayor preparación para el destete. Las madres con niveles de educación más altos y orientación formal sobre lactancia materna demostraron una mayor autonomía en las decisiones sobre la alimentación y dependían menos de las prácticas tradicionales de destete. Sin embargo, el estudio se enfrentó a varias limitaciones. En primer lugar, la Escala de Preparación para el Destete (RWS) se desarrolló originalmente y se validó psicométricamente entre madres turcas, lo que demostró su buena validez y fiabilidad para su uso en esta población. Sin embargo, al igual que con todas las herramientas de medición, una validación adicional en diversas regiones y subgrupos turcos reforzaría aún más su generalizabilidad. En segundo lugar, la dependencia de datos autoinformados puede introducir sesgos de recuerdo y de deseabilidad social. En tercer lugar, aunque se presentaron modelos estadísticos, su poder explicativo fue moderado y es probable que factores no medidos influyan en las decisiones de las madres. Para aprovechar estos hallazgos, las investigaciones futuras deberían considerar enfoques cualitativos para explorar en profundidad las percepciones de las madres sobre la preparación para el destete. Además, se recomiendan estudios multicéntricos que incluyan diversas regiones y poblaciones para mejorar la generalización. Los futuros estudios multicéntricos también deberían completar una validación completa de la RWS guiada por Normas basadas en el consenso para la selección de instrumentos de medición del estado de salud (COnsensus-based Standards for the selection of health status Measurement INstrument -COSMIN) para confirmar la estructura factorial, la invarianza y la capacidad de respuesta en diversos entornos turcos.

## Data Availability

El conjunto de datos que respalda los resultados de este estudio no está disponible públicamente debido a restricciones de privacidad o éticas.
